# Correlated and Anticorrelated Binocular Disparity Modulate GABA+ and Glutamate/Glutamine Concentrations in the Human Visual Cortex

**DOI:** 10.1523/ENEURO.0355-24.2025

**Published:** 2025-03-13

**Authors:** Jacek Matuszewski, Ivan Alvarez, William T. Clarke, Andrew J. Parker, Holly Bridge, I. Betina Ip

**Affiliations:** ^1^Wellcome Centre for Integrative Neuroimaging, Nuffield Department of Clinical Neurosciences, University of Oxford, Oxford OX3 9DU, United Kingdom; ^2^Laboratory of Brain Imaging, Nencki Institute of Experimental Biology, Polish Academy of Sciences, Warsaw 02-093, Poland; ^3^Institut für Biologie, Otto-von-Guericke Universität, Magdeburg 39120, Germany; ^4^Department of Physiology, Anatomy and Genetics, University of Oxford, Oxford OX1 3PT, United Kingdom

**Keywords:** anticorrelation, binocular disparity, fMRS, GABAergic inhibition, lateral occipital cortex, ventral visual cortex

## Abstract

Binocular disparity is used for perception and action in three dimensions. Neurons in the primary visual cortex respond to binocular disparity in random dot patterns, even when the contrast is inverted between eyes (false depth cue). In contrast, neurons in the ventral stream largely cease to respond to false depth cues. This study evaluated whether GABAergic inhibition is involved in suppressing false depth cues in the human ventral visual cortex. We compared GABAergic inhibition (GABA+) and glutamatergic excitation (Glx) during the viewing of correlated and anticorrelated binocular disparity in 18 participants using single-voxel proton magnetic resonance spectroscopy. Measurements were taken from the early visual cortex (EVC) and the lateral occipital cortex (LO). Three visual conditions were presented per voxel location: correlated binocular disparity, anticorrelated binocular disparity, or a blank gray screen with a fixation cross. To identify differences in neurochemistry, GABA+ or Glx levels were compared across viewing conditions. In EVC, correlated disparity increased Glx over anticorrelated and rest conditions, also mirrored in the Glx/GABA+ ratio. In LO, anticorrelated disparity decreased GABA+ and increased Glx. The Glx/GABA+ ratio showed increased excitatory over inhibitory drive to anticorrelated disparity in LO. Glx during viewing of anticorrelation in LO was predictive of object-selective BOLD activity in the same region. We provide evidence that early and ventral visual cortices change GABA+ and Glx concentrations during presentation of correlated and anticorrelated disparity, suggesting a contribution of cortical excitation and inhibition to disparity selectivity.

## Graphical abstract

**Figure:**
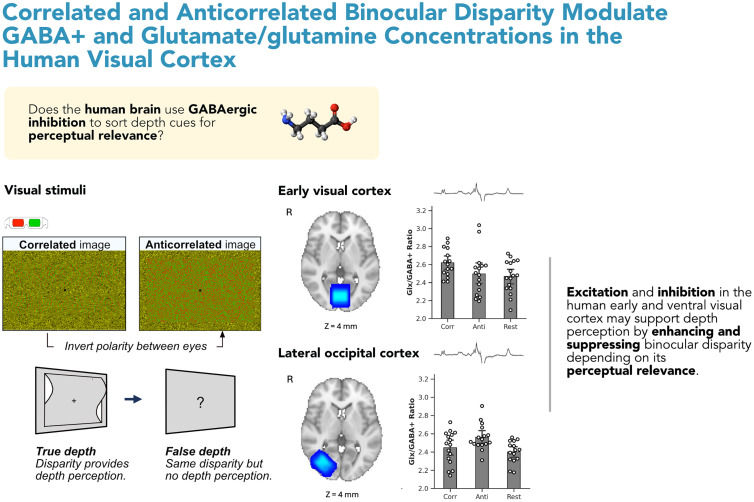
Visual Abstract

## Significance Statement

The visual system must correctly match elements from the left and right eye for proper reconstruction of binocular depth. At the earliest part of binocular processing, false matches can activate depth detectors; however, the activation to false matches is absent in the ventral visual stream. We tested whether GABAergic inhibition contributes to the suppression of false matches in the ventral stream by measuring GABAergic inhibition and glutamatergic excitation in the human visual cortex during presentation of correct and false matches. Correct matches increased excitation in the early visual cortex, and false matches increased excitation and decreased inhibition in the ventral visual cortex. These results suggest a role for excitation and inhibition in distinguishing depth cues for stereoscopic vision.

## Introduction

**Figure 1.**
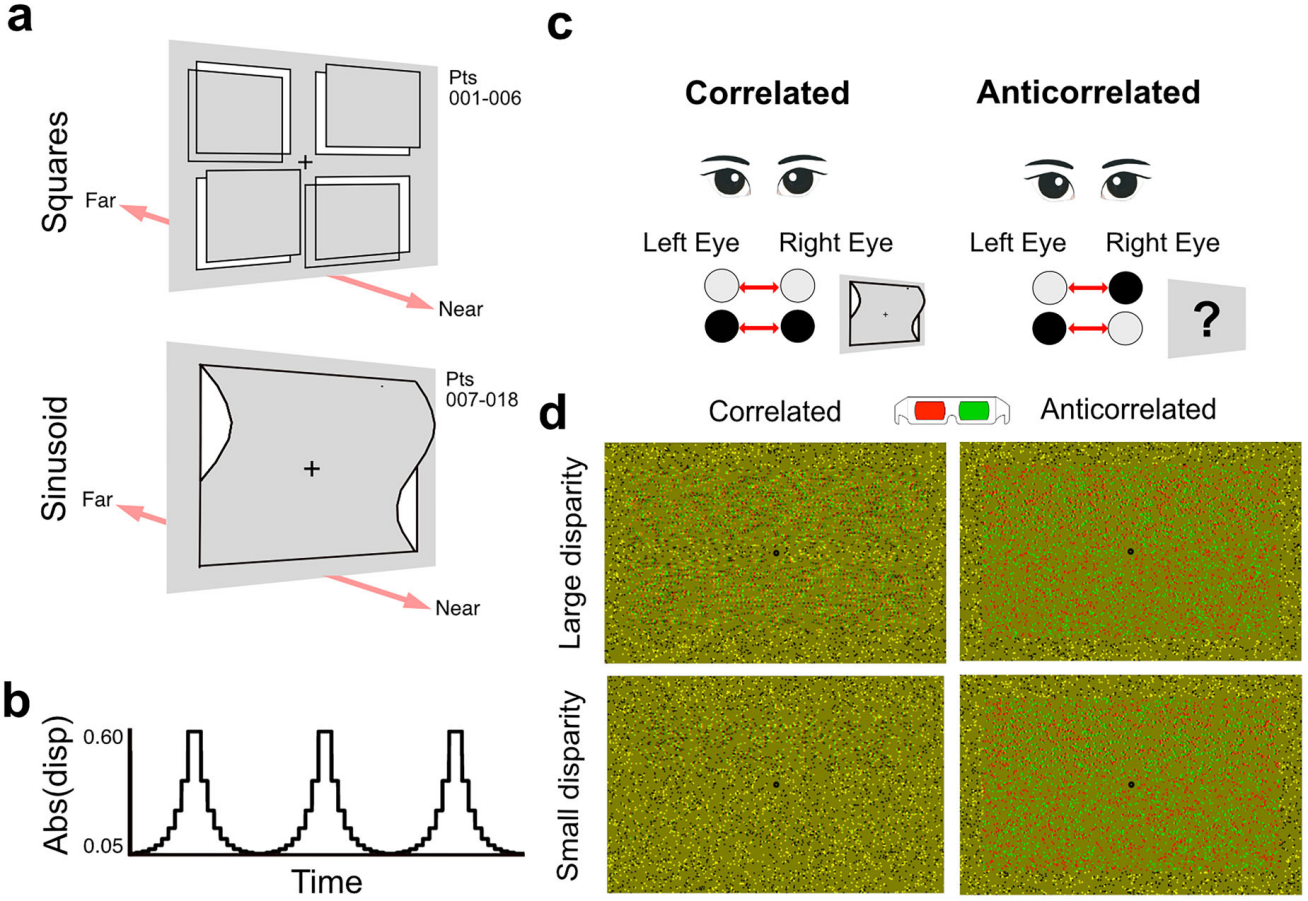
Diagram of random dot stereograms displayed inside the MRI scanner. Two stimulus types were used which were consistent within participants: ***a***, stimuli were composed of dynamic random dot stereograms (RDS) appearing like depth-defined squares for participants (Pts) 001–006, or spatial sinusoids, moving closer and farther in depth to the fixation plane over time, for participants (Pts) 007–018. Disparity type did not affect GABA+ concentrations, and data were pooled together. ***b***, Example maximum absolute disparity in degrees over time. Participants were instructed to monitor the luminance of the RDS stimuli and press a button on a button box when the luminance increased. The main conditions were (***c***) correlated disparity, which presented matching contrast of dots presented to the left and right eye, and anticorrelated disparity, which switched the contrast of the dots. White dots were matched with a black dot and black dots with a white dot. Anticorrelated disparity did not evoke any depth percept. ***d***, Example images of sinusoid RDS in anaglyph, with large disparity amplitude (top, left) and small disparity amplitude (bottom, left). Example anticorrelated images are to the right (top right and bottom right).

The act of seeing requires a transformation of retinal signals to perceptual reality. How the visual system solves the many ambiguities it encounters along the way is an enduring question in neuroscience. A prime example comes from the binocular visual system, where non-matching features in the two eyes create a challenge known as the “correspondence problem,” as elements from each eye need to be matched correctly in the presence of multiple possible false matches (Marr and Poggio, 1979). One experimental approach to investigate the ability of the binocular visual system to eliminate these false matches is to use random dot stereograms (RDS) that carry disparity cues but have opposite contrast in the two eyes (Fig. 1*d*, “anticorrelated” stimuli; Cogan et al., 1993). In V1, disparity-sensitive neurons show an inverted, and attenuated, tuning to anticorrelated compared with correlated RDS (Cumming and Parker, 1997). Since anticorrelated random dot stimuli do not ordinarily result in depth perception (Cumming et al., 1998; with some exceptions, Tanabe et al., 2008), it is thought that visual areas that are sensitive to false matches do not directly play a role in depth perception.

The stereo correspondence problem is solved in the ventral visual cortex of the macaque (Janssen et al., 2003), yet the neural mechanisms by which false matches are suppressed and true matches are facilitated along the visual pathway remain poorly understood. The neuronal response to anticorrelated stimuli decreases along the ventral visual pathway (Kumano et al., 2008) until it ceases in the inferior temporal cortex (Janssen et al., 2003). Ventral visual cortex neurons only respond to true matches that are relevant for perception (Janssen et al., 2000a; Abdolrahmani et al., 2016; Yoshioka et al., 2021), a property that could support depth-invariant object recognition. Hemodynamic responses measured noninvasively from human participants largely support a loss of selectivity to anticorrelation in the lateral occipital area (LO; Bridge and Parker, 2007; Preston et al., 2008), the putative homolog of macaque inferior temporal cortex. In contrast, dorsal extra striate areas MT (Krug et al., 2004) and MST (Takemura et al., 2001) of macaque retain sensitivity to anticorrelated stimuli. These findings suggest that false matches are partially and progressively removed in the ventral visual stream.

How is this suppression accomplished? Several potential and not mutually exclusive physiological mechanisms have been proposed that could account for the disparity selectivity observed (Read and Cumming, 2007). The two main theories (see review by Verhoef et al., 2016) are (1) feedback from more anterior areas in the visual ventral stream, such as area IT, that have a solution for the correspondence problem, or (2) refinement of selectivity through local inhibition in V1. A purely feedback-based mechanism is not supported by the literature. The time course of V4 neurons to anticorrelated disparity is not consistent with a feedback mechanism, as attenuation is instantaneous rather than delayed, as would be expected if a purely feedback-based mechanism was involved (Tanabe et al., 2004). Modifying the binocular energy model with feedforward local processing can account for the observed anticorrelated response in V1 (Read et al., 2002) and therefore support a role for cortical inhibition. Disparity tuning could result from a combination of excitatory and inhibitory activity, as V1 neurons partially suppress false matches, caused by either inhibition or loss of excitation through prior inhibition (Tanabe et al., 2011; Tanabe and Cumming, 2014). Invoking a model with inhibition from local interactions additionally accounts for further anticorrelated disparity responses in V1 (Samonds et al., 2013). Adaptation to anticorrelated disparity increased the EEG measure of visual excitation, possibly signaling larger inhibitory activity for processing anticorrelated disparity in the visual cortex (Rideaux et al., 2020). Overall, these studies provide indirect evidence suggesting that GABAergic inhibition, which plays a role in neural suppression, may be involved in the processing of anticorrelated disparity. No study has directly probed GABAergic inhibition in relationship to anticorrelated disparity processing.

We measured GABAergic inhibition using the GABA+ signal from the early visual cortex (EVC) and the lateral occipital cortex (LO) in the human brain. Participants viewed correlated and anticorrelated binocular disparity during measurements. We also measured signals relating to glutamatergic excitation, using the glutamate + glutamine complex (Glx). Our results showed surprisingly that viewing of anticorrelated stimuli decreased inhibition and increased excitation in ventral area LO. A similar pattern was not present in the early visual cortex. These results are considered in relation to the stages of processing of binocular information in the human visual cortex.

## Materials and Methods

### Participants

Eighteen participants (13 females; mean = 29; SD = 8 years) took part in the experiment. All had normal, or corrected-to-normal, vision and demonstrated normal stereo acuity using clinical screening plates (<120 arcsecs, TNO Stereo test, Lameris). They had no self-identified neurological impairments. Before recruitment, all participants were verbally screened during the eligibility assessment for drug use and consumption of no more than three cigarettes per day. Each volunteer took part in a 2 h MRI session. A reimbursement of £40 was received for the MRI sessions. All volunteers gave informed and written consent, as approved by the University of Oxford Research Ethics Committee (R54411/RE001). We did not perform an a priori power calculation for the study.

### Experimental setup

A custom-made Wheatstone MRI-stereoscope was used for dichoptic presentation inside the MRI scanner (Ip et al., 2022). Inside the scanner, participants verbally confirmed seeing stereoscopic depth during a brief calibration procedure prior to data collection. Participants viewed stimuli on a γ-linearized LCD screen (BOLDscreen 32, Cambridge Research Systems, viewing distance = 127.5 cm). The dichoptic display size in degrees of visual angle was 10.23° × 12.88°. All participants wore transparent protective goggles with or without MRI-safe visual correction.

### Visual stimuli

Figure 1 shows the experimental conditions. Two different disparity-defined stimuli were used but kept consistent within participant (Fig. 1*a*, top): six participants were presented with a rectangular patch in which the peak disparity of four small quadrants varied between ±0.3° in logarithmic steps in depth without going through ±0.05°. The top right and bottom left quadrant were matched in disparity, and the top left and bottom right quadrant were matched in equal and opposite disparity. The stimulus was changed to a sinusoid to further optimize presentation for the lateral occipital cortex. The remaining 12 participants were presented with a RDS stimulus appearing like a spatial sinusoid (Fig. 1*a*, bottom), in which the peak disparity varied between ±0.6° in logarithmic steps in depth without going through ±0.1°. In six out of these 12 participants, the stimulus briefly appeared completely flat before transitioning to ±0.1°. Stimuli were presented continuously during each MRS condition without any rest. A linear mixed model showed that the stimulus type (“square”, “sinusoid”) had no effect on spectral quality or GABA+ concentration and conditions were analyzed together. RDS filled the entire display screen and consisted of 5,000 dots (50% white and 50% black; dot radius, 0.05°; dot refresh rate, 30 Hz). Stimuli subtended 7.23° × 9.88° and were framed by a 1.5° correlated, zero disparity border. A central fixation dot (black, 0.4°) was present on each frame, surrounded by a dot-free zone (0.8°) to prevent vergence eye movements caused by changing disparities at fixation. The disparity in the RDS was modulated from positive to negative disparity over time to reduce adaptation effects by cycling six times over the MRS acquisition runtime (Fig. 1*b*). Anticorrelated RDS were identical to correlated RDS (Fig. 1*c*), with the exception that the interocular contrast of the dots was inverted and no perception of depth was reported (Fig. 1*d*). During correlated and anticorrelated runs, participants looked at the fixation dot and performed an easy contrast detection task. Contrast differences occurred at random points during the run (72 times/run, 1 s in duration) and comprised a 20% linear decrease in contrast across the stimulus area. A similar task was used in Alvarez et al. (2025), and analysis of these data showed no difference in performance between correlated and anticorrelated stimuli. In the current study data from button presses was not recorded due to a Matlab error. The rest condition was a mid-gray screen with a fixation dot. Participants were asked to fixate, and no task was provided. We collected six MRS runs per participant (rest, correlated, and anticorrelated conditions in both EVC and LO). Across participants, half had EVC data collected first and the other half LO collected first. Within each of the ROIs, the presentation order of rest, correlated and anticorrelated disparity was randomized. Each MRS acquisition lasted a total of 8 min and 13 s, including dummy scans.

### MRI data acquisition

Magnetic resonance images were acquired using a 3 T Siemens Prisma (Siemens Healthineers), using a 64-channel head and neck coil. A 1 mm isotropic whole-head T1-weighted image was collected for placement of the spectroscopic volume of interest (MPRAGE; TR, 1,900 ms; TE, 3.97 ms; field of view, 192 × 192 mm^2^; 192 slices; flip angle, 8°), with a total acquisition time of 5 min 31 s. To locate the lateral occipital cortex region, an object localizer scan was performed prior to MRS voxel placement (2.4 mm isotropic resolution; MB8; TR, 1,000 ms; TE, 39 ms; 66 slices; flip angle, 52°). Stimuli were presented in a block design, with intact objects (15 s) alternating with phase-scrambled objects (15 s) that preserved the same contrast and phase information as intact objects, while removing shape information. The total acquisition time was 3 min 10 s. A fixation cross was always present, and each trial was presented for 1,000 ms (800 ms stimulus followed by 200 ms mid-gray screen). Participants were instructed to passively fixate during the localizer experiment. The raw MRI data can be accessed at https://zenodo.org/records/14290083.

### MRS procedure

Single-voxel MRS data were acquired using a MEGA-PRESS sequence, consisting of a locally developed version of the CMRR spectroscopy package MEGA-PRESS sequence (Willis et al., 2023). Acquisition parameters consisted of the following: voxel size, 20 × 25 × 25 mm^3^ (short dimension in the medial-lateral direction for LO, and with the long dimension aligned with the tentorium cerebelli for EVC); echo time (TE), 68 ms; repetition time (TR), 1,500 ms; number of spectra, 320 (160 edit-on and 160 edit-off spectra); VAPOR and dual-band editing pulse water suppression; 22.3 ms editing pulse for a 53 Hz bandwidth, which was centered at 1.9 ppm (edit-on condition) and at 7.5 ppm (edit-off condition) in alternation; 16-step phase cycling; a total of 8 min 13 s run time. A water unsuppressed reference was collected after each condition to provide an internal reference.

**Figure 2.**
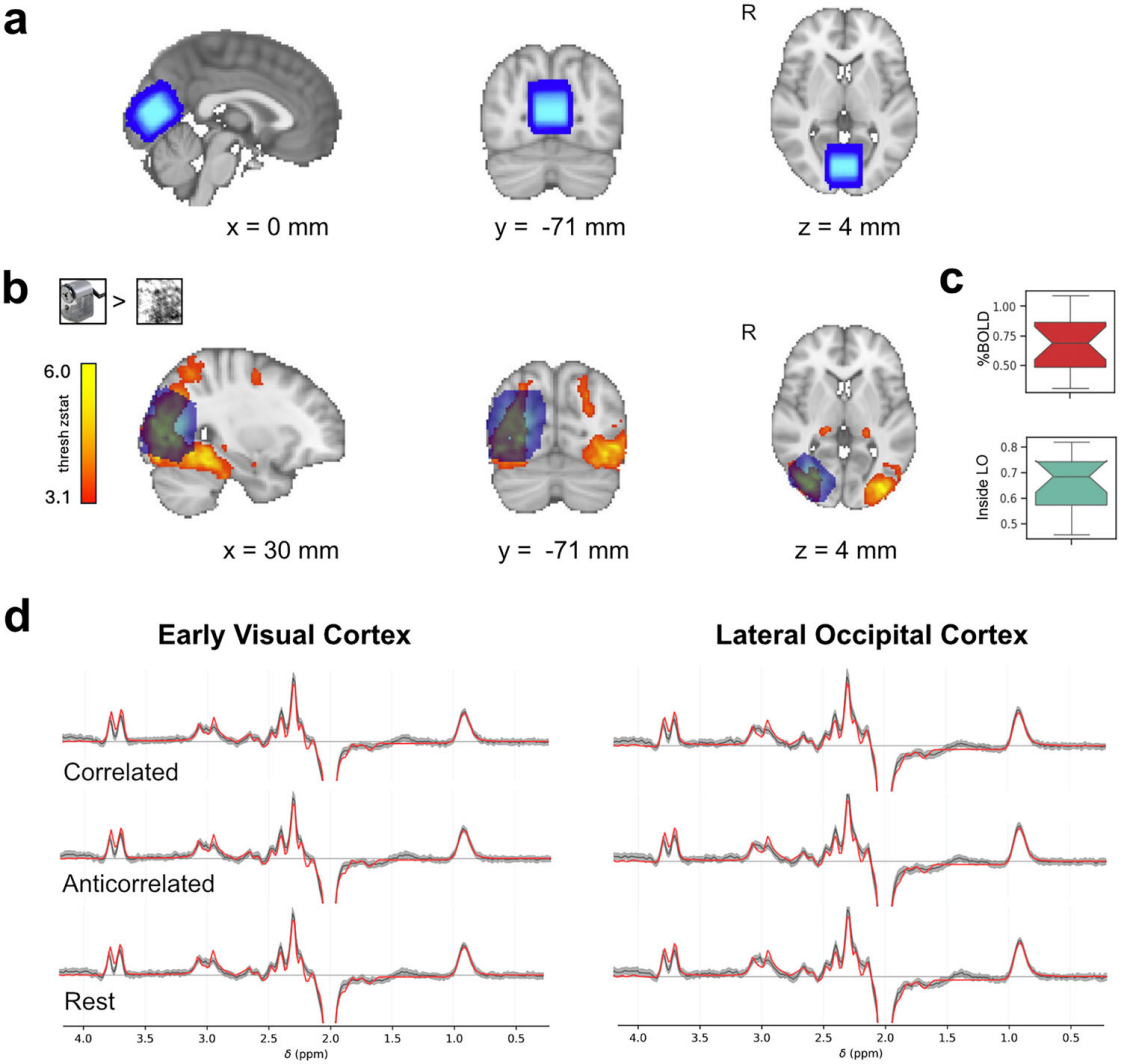
MRS voxel in early and ventral visual cortex and group spectra for each condition. ***a***, Proton magnetic resonance spectroscopy (MRS) voxel locations for all 18 participants positioned in the early visual cortex (EVC; blue) and (***b***) right lateral occipital cortex (LO; blue) displayed on the MNI152 2 mm standard brain. ***c***, Boxplots show %BOLD change to objects inside the LO voxel and proportion overlap between statistical activation maps to objects and the LO voxel. ***d***, Group MRS spectra for each condition and voxel, including only data that was used for the main GABA+ analysis. The black line represents the mean across participants, the gray area shows ±1 standard deviation across participants, and the red line shows the model fit. Spectral amplitude is scaled to arbitrary units. *x*-axis shows chemical shift in parts per million. Slice positions are indicated in MNI coordinates. The boxplot shows the central 50% range of the data, the whiskers span 1.5 the interquartile range from the upper and lower quartile. R, right hemisphere.

The region of interest for EVC was first centered on the occipital midline to cover equivalent portions of the right and left visual cortex, then angled to be parallel to the calcarine sulcus, and moved as posterior as possible while avoiding contamination by the cerebellar tentorium and the sagittal sinus (Fig. 2*a*). For LO placement, the voxel was positioned in the right lateral occipital cortex using the object localizer. The right hemisphere has been shown to respond more to disparity than the left (Ip et al., 2014; Lohia et al., 2024). Object localizer data were analyzed on-line using dynamic *t*-maps of the contrast between objects and phase-scrambled objects. Contrasting the intact with phase-scrambled objects isolated regions where hemodynamic responses increased to intact objects, as shown by activation clusters in bilateral ventral visual cortex (Fig. 2*b*). In confirmation of the accuracy of the voxel placement, we found substantial %BOLD change to objects inside the LO voxel (Fig. 2*c*, top; *n* = 18, mean = 0.68 ± 0.23) and 0.66 proportion overlap between the LO voxel with the statistical maps from the object localizer (Fig. 2*c*, bottom; *n* = 18, mean = 0.66 ± 0.10). Group spectra were constructed using fsl_mrs_summarise. Only data used for GABA+ analysis, after outlier rejection, were included in group spectra (Fig. 2*d*).

### fMRI analysis

For each subject, functional data from the object localizer were preprocessed and analyzed with FSL FEAT (version 6.0.7.10; http://www.fmrib.ox.ac.uk/fsl). This pipeline included realignment and head movement correction (MCFLIRT), registration to MNI152 standard space (2 mm resolution), high pass filtering (30 s cutoff), and spatial smoothing with 5 mm Gaussian full-width at half-maximum (FWHM) kernel. Next, timings of intact and phase-scrambled object conditions and six standard head motion parameters were entered into subject-specific first-level general linear models (GLM). Finally, “object > scrambled” contrasts from all subjects were entered into a group-level mixed effects analysis (FSL FLAME 1). Group-level maps were cluster corrected for multiple comparisons with a *Z* threshold of 3.1 (*p* < 0.05) and overlapped with the group mask of the LO MRS voxel created by joining all participants' masks. Additionally, to investigate BOLD signal responses in the LO MRS voxel location, we computed intersections between each participant's functional map and their LO MRS voxel with fslmaths. Then, we used featquery to calculate the mean BOLD change (%) in the obtained mask.

### MRS data analysis

Data analysis was performed using the open-source toolbox FSL-MRS v.2.1.19 (Clarke et al., 2021). Processing included conversion from the vendor .dat (or TWIX) format to NIfTI format using spec2nii v.0.7.4 (Clarke et al., 2022); preprocessing included the following: coil-combination, windowed averaged phase and frequency alignment between repeats, eddy current correction, truncation of the FID to remove two time-domain points before the echo center, removal of residual water peak using Hankel Lanczos singular value decomposition (HLSVD) over 4.5–4.8 ppm, and phase and frequency alignment between averaged edit-on and edit-off spectra. A phase-corrected, non-water-suppressed reference was also generated during preprocessing from integrated reference scans. Model fitting was performed using a linear combination model: basis spectra were fitted to the complex-valued spectrum in the frequency domain by scaling, shifting, and broadening them. Then, basis spectra were divided into two metabolite groups, with macromolecular peaks allowed to broaden and shift independently of other metabolites. The model fitting was achieved using the truncated Newton algorithm as implemented in SciPy. A 0th-order complex polynomial baseline was fitted concurrently. To model metabolites in the “edit-on” minus “edit-off” difference spectrum, we used a simulated basis set containing the model spectra for *N*-acetylaspartate (NAA), *N*-acetylaspartylglutamate (NAAG), γ-aminobutyric acid (GABA), glutamine (Gln), glutamate (Glu), glutathione (GSH), macromolecules (MM), and combined NAA + NAAG, Glu + Gln + GSH, and GABA+ MM (https://git.fmrib.ox.ac.uk/wclarke/win-mrs-basis-sets). As the GABA signal at 3.0 ppm contains coedited macromolecule (MM) signals, as well as homocarnosine (Rothman et al., 1997), the combined GABA + MM signal is reported and referred to as GABA+. The combination of Glu + Gln + GSH is referred to as Glx. Both Glx and GABA+ were extracted from the difference spectra. Absolute concentrations in millimole per kilogram (mMol/kg), corrected for tissue fraction and tissue relaxation are reported. FSL FAST was applied to the T1-weighted image for voxel tissue type fraction estimation, separating white matter, gray matter, and cerebrospinal fluid, for tissue fraction correction.

### MRS data quality measure

**Figure 3.**
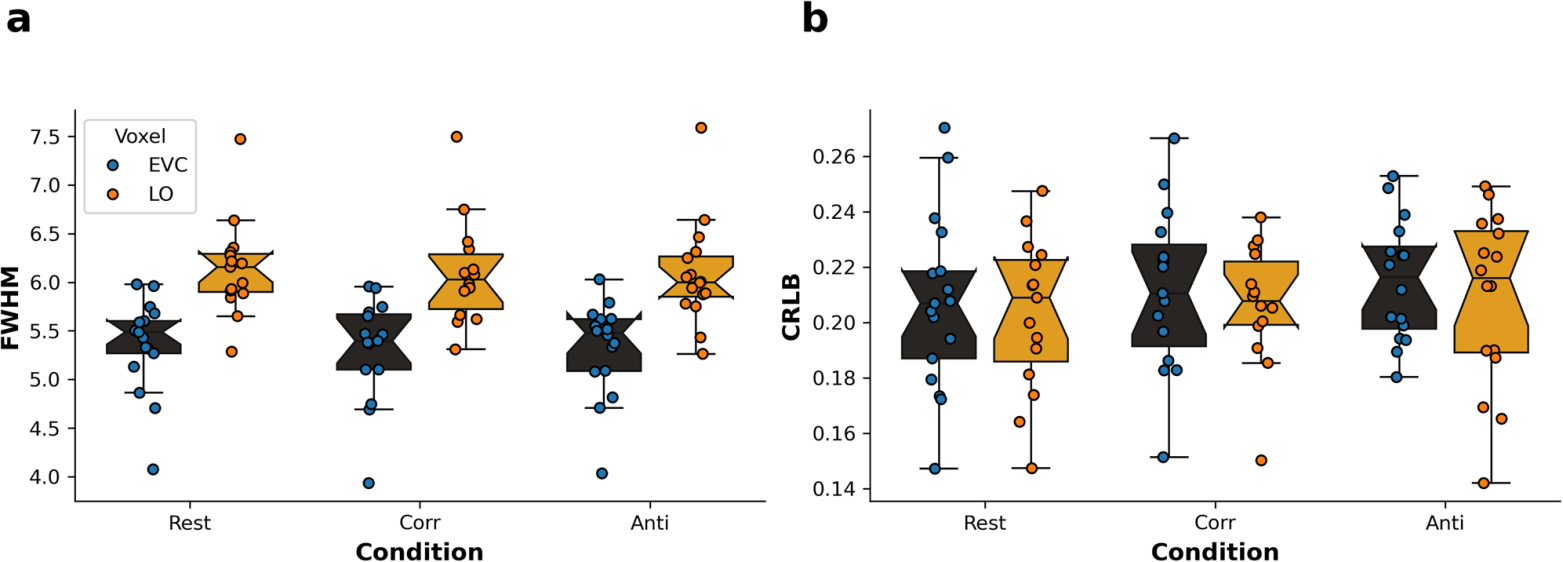
MRS quality measures in early visual cortex (EVC) and lateral occipital cortex (LO). Boxplots show spectral quality measure NAA linewidth by voxel and condition (***a***) and goodness of model fit measure absolute Cramér–Rao lower bounds (CRLB) for GABA+ by voxel and condition (***b***). EVC, black boxplot, blue dots; LO, orange boxplot, orange dots. The boxplot shows the central 50% range of the data, the whiskers span 1.5 of the interquartile range from the upper and lower quartile. Corr, correlated condition; Anti, Anticorrelated condition; FWHM, full-width at half-maximum.

Prior to summary statistics, an outlier analysis based on metabolite concentrations was performed to ensure that data represented the norm. GABA+ and Glx concentrations outside of the 1.5 times interquartile range were excluded. Some conditions could not be collected from all participants, and MRS quality control excluded some individual data points. We ended up with a minimum of 15 participants per condition. The spectral quality of the acquisition was determined using the linewidth of the full-width at half-maximum (FWHM) of the inverted *N*-acetylaspartate singlet at 2.01 ppm and reported in Hz. The GABA+ estimation of model fit was reported as absolute Cramér–Rao lower bounds (CRLB). We assessed MRS data quality across voxel locations and conditions. There was no interaction effect of voxel and condition on NAA linewidth (*F*_(1,77.275)_ = 0.42, *p* = 0.66), meaning that acquisition quality did not depend on visual condition. A model without interaction showed a highly significant effect of voxel on spectral quality (Fig. 3*a*; *F*_(1,79.12)_ = 183.73, *p* < 2 × 10^−16^), with LO linewidth broader than EVC. No effects of condition were found (*F*_(1,79.20)_ = 0.079, *p* = 0.92). In contrast, no effect of voxel or condition was found on the quality of the GABA+ model fit (Fig. 3*b*; *p* > 0.05). Based on the linewidth difference, we performed separate analyses for EVC and LO voxels as they could not be directly compared and added NAA linewidth as a covariate of no interest in the LMM analyses. We also controlled for linewidth in the partial correlations.

We compared the correlation between GABA+ and Glx across conditions and within each voxel location for test–retest reliability using data that contributed to the main analysis. First, the Pearson's correlation coefficient between the first and second half of the run was calculated by metabolite (GABA+, Glx) and by the voxel location (EVC, LO). Then, we applied a two-tailed Fisher's *r*-to-*z* transform test to evaluate the significance of the difference between correlation coefficients across conditions and within each voxel location. Correlation coefficients were very strong within metabolites for the early visual cortex (GABA+, *r*_(16)_ = 0.86, *p* < 0.001; Glx, *r*_(16)_ = 0.97, *p* < 0.001) and the lateral occipital cortex (GABA+, *r*_(16)_ = 0.80, *p* < 0.001; Glx, *r*_(16)_ = 0.98, *p* < 0.001). When comparing across metabolites, we found a significant difference in correlation coefficients between GABA+ and Glx in LO (*p* = 0.001) and a marginally significant difference in EVC (*p* = 0.03). This is consistent with the lower concentration of GABA+ relative to Glx. When comparing across voxels, we found no difference in correlation coefficients for either GABA+ (*p* = 0.60) or Glx (*p* = 0.56).

### Statistical analysis

Data analysis was conducted using RStudio (RStudio Version 2023.06.0+421). Metabolite concentrations were analyzed with mixed effects linear models computed with LME4 R package (Bates et al., 2015). The model syntax was constructed to test for fixed effects of condition within voxel, including the dummy variable “spectral quality” and the random effect of “participant.” Separate analyses were performed for each voxel (EVC, LO) and each metabolite (GABA+, Glx). A model without interaction was used if no interaction effects were found. The ANOVA function was used to obtain *p* values. A type II analysis of variance table with Kenward–Roger's method was used when no interactions were present. Each linear model was performed with an expression: “Metabolite Concentration ∼ Condition + NAA FWHM + (1|Participant)”. If a fixed effect was significant, post hoc analyses were performed using Tukey method for multiple comparisons of means for parametric models with *p* values adjusted using Bonferroni–Holm correction (R, multcompare): summary(glht(model, linfct = mcp(cond = “Tukey”)), test = adjusted(“holm”)). Pearson's correlations coefficients with and without partial correlations were computed with pingouin (Vallat, 2018) to assess the relationship between GABA+ and Glx in each voxel and condition. The excitation/inhibition index was calculated by dividing Glx/GABA+ using the same data points as for the individual metabolite analyses. We applied two-tailed Fisher's *r*-to-*z*-transform tests (http://vassarstats.net/rdiff.html) to test the significance of the difference in correlation coefficients.

## Results

We evaluated if GABA+ in the early or ventral visual cortex was modulated by presenting correlated random dot stereograms (RDS) or anticorrelated RDS, which have identical disparity magnitudes but inverted contrast between eyes. As a baseline, we also presented a rest condition that required fixation on a mid-gray screen. Our hypothesis was that ventral visual cortex GABA+ levels would increase to anticorrelated disparity, whereas no change was expected in the EVC. We expected an increase in Glx during correlated and anticorrelated conditions compared with the rest condition, as a positive control for functional changes in glutamatergic excitation during visual stimulation. The summary metric Glx/GABA+ was calculated to evaluate changes in excitation and inhibition balance. In exploratory analyses, the relationship between LO GABA+ and Glx and the hemodynamic response to objects was assessed. Finally, metabolite concentrations from the early and ventral visual cortex allowed us to investigate the balance between GABA+ and Glx at two different stages of the visual processing hierarchy.

### Disparity correlation modulates GABAergic inhibition (GABA+) and glutamatergic excitation (Glx) in the early and ventral visual cortex

**Figure 4.**
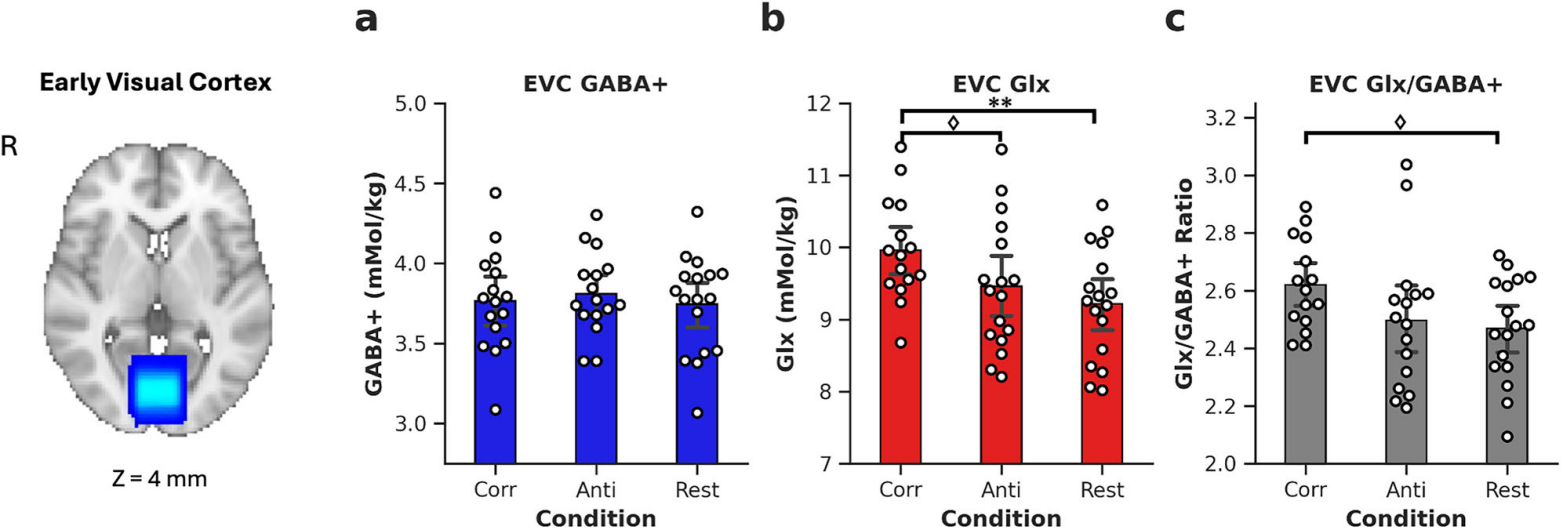
Bar plots show concentrations of metabolites for visual conditions for the early visual cortex. GABA+ (***a***), Glx (***b***), and Glx/GABA+ (excitation/inhibition) ratio (***c***). Dots show individual participants. Error bars are ±1 SD. Bonferroni–Holm adjusted *p* values, *<0.05, **<0.01; marginal significance ♢<0.1. Voxels show overlap across 18 participants. R, right hemisphere; EVC, early visual cortex; GABA+, γ-aminobutyric acid + macromolecules; Glx, glutamate + glutamine; Corr, correlated condition; Anti, anticorrelated condition. Slice positionis indicated in MNI coordinates.

Viewing different stimulus conditions had no effect on GABA+ concentration in the EVC (Fig. 4*a*; EVC, *p* = 0.64). When investigating glutamatergic excitation (Glx), we found that there was a significant effect of viewing conditions in the EVC (Fig. 4*b*; *F*_(2,29.38)_ = 5.267, *p* = 0.011). Specifically, seeing the correlated stimulus marginally increased Glx over anticorrelated, *p* = 0.071, and over rest, *p* = 0.004. The excitation/inhibition ratio (“E/I ratio”) evaluated the overall excitatory drive by dividing Glx by GABA+ (Fig. 4*c*). Viewing conditions marginally modulated E/I ratio in the EVC (*F*_(2,30.18)_ = 2.87, *p* = 0.072). Specifically, viewing correlated stimuli increased E/I ratio in the EVC relative to rest (*p* = 0.067).

**Figure 5.**
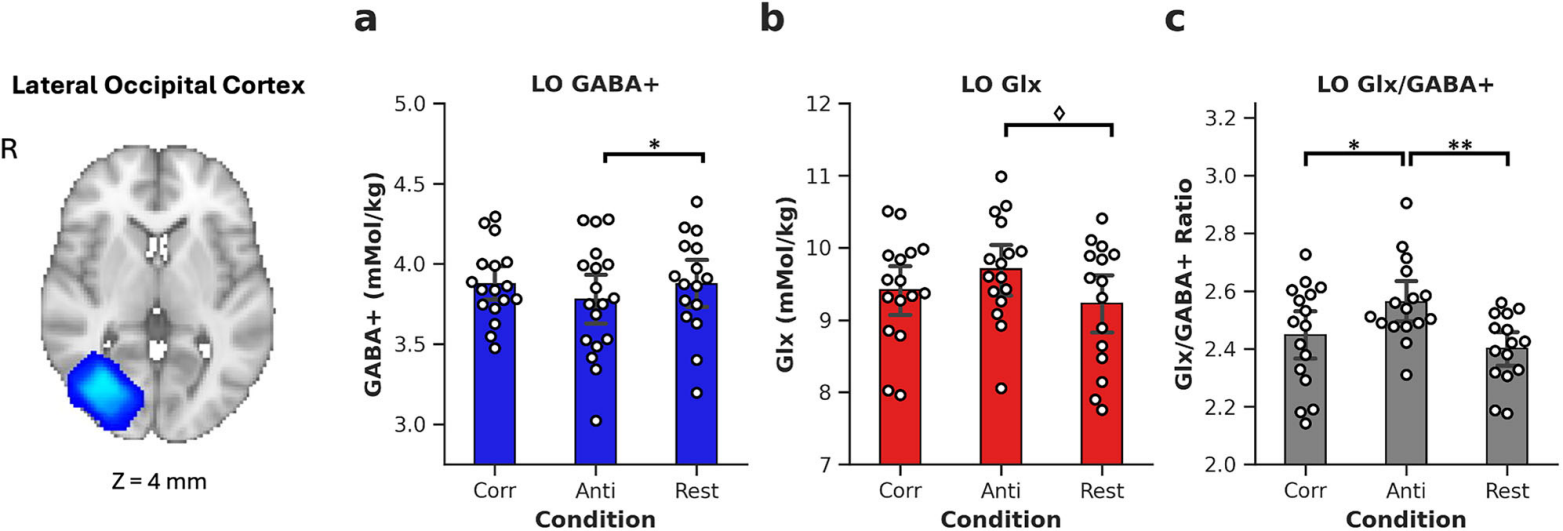
Bar plots show concentrations of metabolites for visual condition for the lateral occipital cortex. GABA+ (***a***), Glx (***b***), and Glx/GABA+ (excitation/inhibition) ratio (***c***). Dots show individual participants. Error bars are ±1 SD. Bonferroni–Holm adjusted *p* values, *<0.05, **<0.01; marginal significance ♢ < 0.1. Voxels show overlap across 18 participants. R, right hemisphere; LO, lateral occipital cortex; GABA+, γ-aminobutyric acid + macromolecules; Glx, glutamate + glutamine; Corr, correlated condition; Anti, anticorrelated condition. Slice positions are indicated in MNI coordinates.

Viewing conditions modulated GABA+ in LO (Fig. 5*a*; *F*_(2,30.58)_ = 3.42, *p* = 0.047); viewing anticorrelation lowered GABA+ compared with rest (*p* = 0.027). This was contrary to our hypothesis that GABA would increase during presentation of disparity anticorrelation. In LO, viewing anticorrelation marginally increased Glx compared with rest (Fig. 5*b*; *p* = 0.055). Viewing condition influenced the E/I balance in LO (*F*_(2,29.1)_ = 6.21, *p* = 0.006); viewing anticorrelation increased E/I balance over correlation (*p* = 0.018) and over rest (*p* = 0.002).

In this section, we demonstrated that correlated binocular disparity increased cortical excitation in the early visual cortex, whereas anticorrelated disparity increased excitation in the ventral visual area LO. Excitatory drive in LO increased during anticorrelated disparity compared with correlated disparity or rest.

### Glutamatergic excitation (Glx) and GABAergic inhibition (GABA+) correlate in early and ventral visual cortex

The ratio of Glx/GABA is a proxy for excitatory and inhibitory balance for noninvasive brain imaging. The balance between excitation and inhibition is thought to enhance neuronal selectivity and support plasticity. In the human brain, their correlation has been shown to vary by brain region (Steel et al., 2020; Rideaux, 2021). While E/I balance has been studied in the early visual cortex, little is known about E/I balance in the ventral visual cortex. Here we aimed to assess if their balance differed by disparity (defined by viewing condition) and by voxel location.

**Figure 6.**
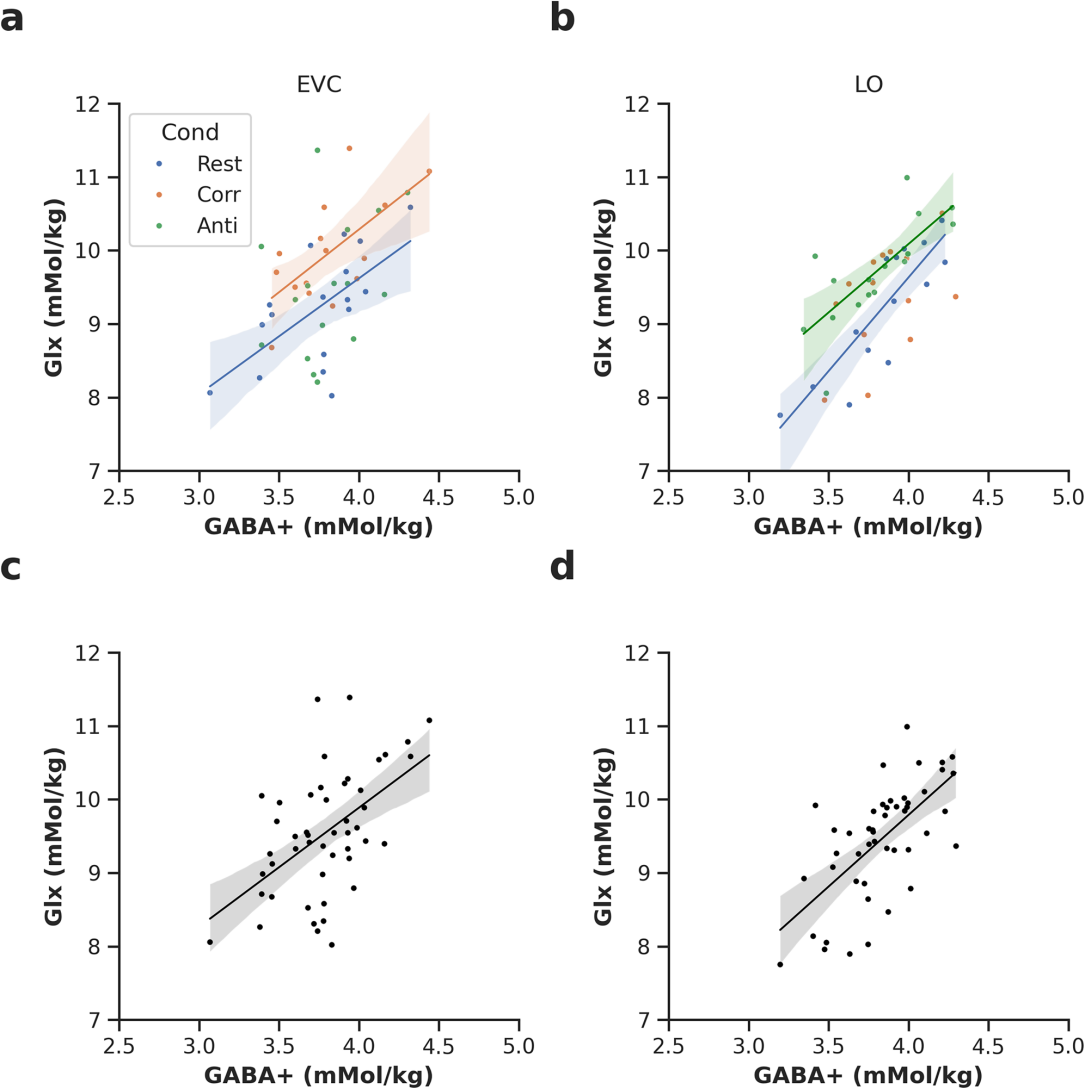
Glx and GABA+ were correlated within conditions for EVC and LO. Correlations between Glx and GABA+ for EVC (***a***) and for LO (***b***). Pooled across conditions for EVC (***c***) and LO (***d***). Model fit is plotted where *p* < 0.05 uncorrected. Colors indicate condition; red, correlated (Corr); blue, rest; green, anticorrelated (Anti); black, pooled across conditions. GABA+, γ-aminobutyric acid + macromolecules; Glx, glutamate + glutamine.

In EVC, we found that Glx and GABA+ were moderately correlated (Fig. 6*a*; Rest, *r* = 0.63, BF_10_ = 8.44, *p* = 0.007; Corr, *r* = 0.65, BF_10_ = 7.12, *p* = 0.009). Only the anticorrelated condition did not show a significant correlation (*r* = 0.35, BF_10_ = 0.66, *p* = 0.206). In LO, we found weak evidence for a correlation between Glx and GABA+ in the correlated condition (*r* = 0.49, BF_10_ = 1.39, *p* = 0.076), but strong evidence (Fig. 6*b*; Anti, *r* = 0.72, BF_10_ = 15.47, *p* < 0.001; Rest, *r* = 0.85, BF_10_ = 403.63, *p* < 0.001) for this correlation for the anticorrelated and rest conditions, respectively. Overall, the trend across conditions was positive, so data were pooled across conditions to investigate the E/I balance across the two voxel locations. The correlation was extremely high in both EVC (Fig. 6*c*; *n* = 48, *r* = 0.53, BF_10_ = 230.227, *p* = 0.0001) and LO (Fig. 6*d*; *n* = 47, *r* = 0.65, BF_10_ = 6.83 + 04, *p* < 0.0001). These correlations were robust to controlling for several confounding factors separately, including spectral quality, participant age, sex, and GABA+ model fit in EVC and LO. However, the correlation in EVC was not robust to controlling for Glx model fit, whereas it remained robust in LO (Table 1). When controlling for all confounding factors in the same model, the correlation between Glx and GABA+ was no longer significant for EVC (*r* = 0.214, *p* = 0.169), and the correlation in LO remained significant (*r* = 0.392, *p* = 0.0124). We assessed the significance of the difference in correlation coefficient between voxels using a Fisher's *r*-to-*z* transform and found none (*p* = 0.358).

**Table 1.** Partial correlation while controlling for confounding factors for the early visual cortex (EVC) and lateral occipital cortex (LO)

Controlling for	EVC		LO	
	*r*	Corrected *p* value	*r*	Corrected *p* value
Spectral quality	0.53	0.0005***	0.54	0.0005***
Age	0.51	0.0015**	0.63	<0.0001****
Sex	0.48	0.003**	0.65	<0.0001****
GABA+ fit error	0.39	0.034*	0.45	0.00195**
Glx fit error	0.30	0.209 NS	0.60	<0.0001****
Spectral quality, Age, Sex, GABA+ fit error and Glx fit error	0.214	0.169 NS	0.392	0.0124*

*p* values were Bonferroni corrected for five multiple comparisons except for the full model. GABA+, γ-aminobutyric acid + macromolecules; Glx, glutamate + glutamine + glutathione. *p < 0.05, **p < 0.01, ***p < 0.001, ****p < 0.0001, NS, not significant.

These results show a tight balance of excitation and inhibition in the ventral visual cortex that was not driven by confounding factors.

### The correlation between object-selective BOLD responses and glutamatergic excitation in the ventral visual cortex

**Figure 7.**
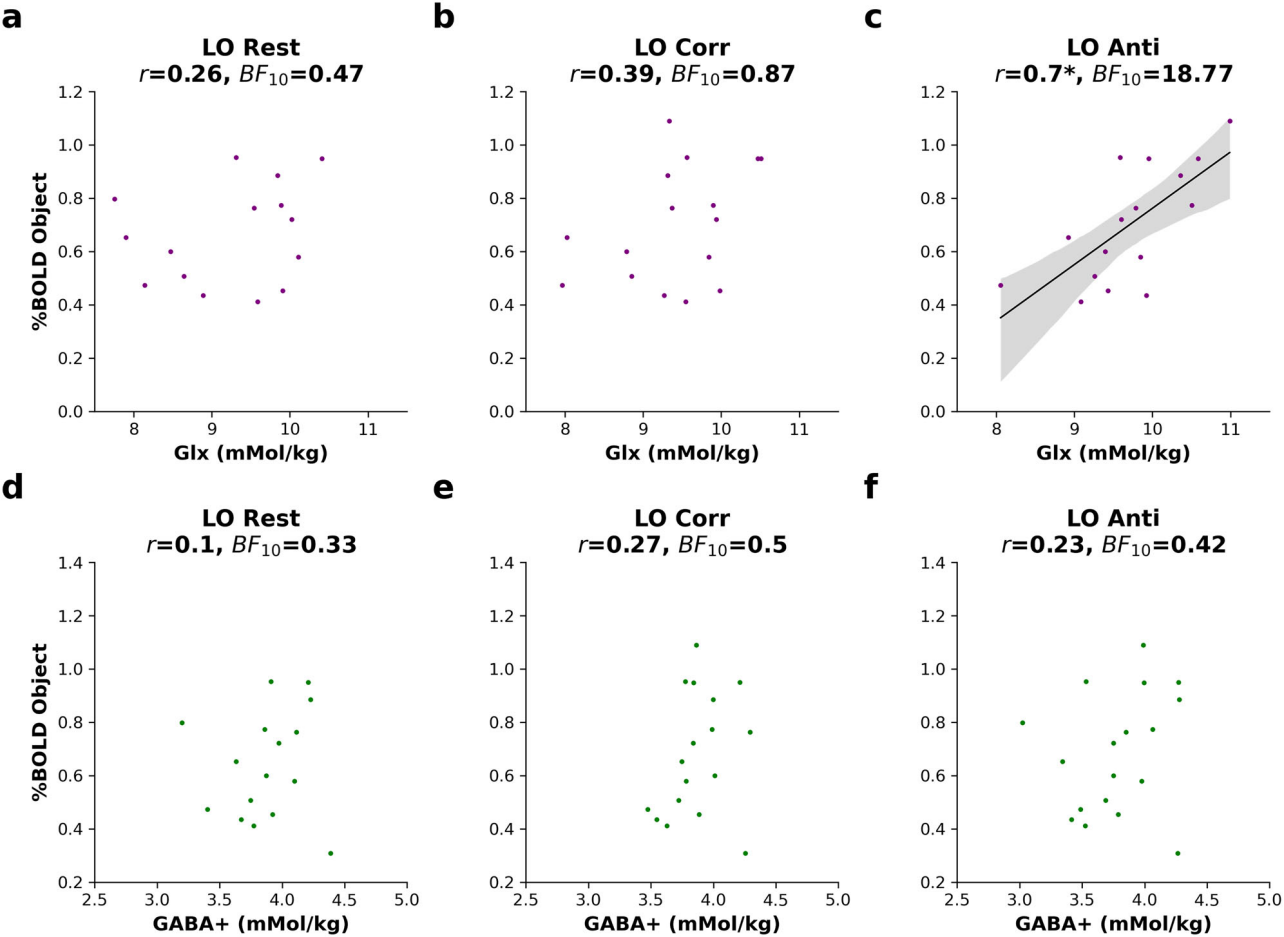
Correlation between %BOLD change to objects with Glx and GABA+ in the lateral occipital voxel. Plots show correlation between %BOLD change to objects with Glx during (***a***) rest, (***b***) correlated (Corr), and (***c***) anticorrelated (Anti) disparity in LO. Below, plots show the correlation between %BOLD change to objects with GABA+ during (***d***) rest, (***e***) correlated, and (***f***) anticorrelated disparity in LO. Model fit is plotted where *p* < 0.05, Bonferroni corrected. LO, lateral occipital cortex; GABA+, γ-aminobutyric acid + macromolecules; Glx, glutamate + glutamine + glutathione; BF_10_, Bayes factor in favor of the alternative hypothesis. * *p *< 0.05, Bonferroni corrected.

Neurons in the ventral visual cortex exhibit two-dimensional (Kourtzi and Kanwisher, 2000; Decramer et al., 2019) as well as three-dimensional (Janssen et al., 2000b; Decramer et al., 2019) object selectivity. The relationship between 2D and 3D object processing in LO is not well understood. In this section, we evaluated the relationship between excitation and inhibition during binocular disparity presentation in LO and 2D object selectivity. Using data from the LO voxel, we correlated the 2D object-selective BOLD responses (intact > scrambled objects) with neurochemistry during continuous viewing of 3D stimuli (Fig. 1*c*). Strong evidence was found for a correlation between object-selective BOLD change and Glx only during viewing of anticorrelated disparity (Fig. 7*c*; *n* = 16, *r* = 0.7, BF_10_ = 18.77, *p* = 0.003). The correlation between LO anticorrelated Glx and BOLD change was robust to controlling for spectral quality (*r* = 0.60, *p* = 0.017). To rule out the possibility that the BOLD activity could have been higher in regions where there was more overlap between the LO voxel with the BOLD activity, we performed partial correlations controlling for percentage overlap. The correlation remained significant after controlling for percentage overlap (*r* = 0.67, *p* = 0.006). To evaluate if general excitability influenced the correlation, we controlled for EVC anticorrelated Glx concentrations and found no weakening of the relationship, *r* = 0.76, *p* = 0.002. The uncorrected correlation was significant after Bonferroni’s correction for six multiple comparisons (*p* = 0.018). No comparable association was present for EVC Anti (*r* = 0.31, *p* = 0.24, BF_10_ = 0.575).

In summary, this section supplied strong evidence for a correlation between object-selective BOLD activity and glutamatergic excitation during viewing of anticorrelated disparity, with greater BOLD activity relating to more excitation. The correlation was robust to controlling separately for spectral quality, percentage overlap, and Glx in the early visual cortex.

## Discussion

Using proton MR spectroscopy in humans, we measured GABAergic inhibition and glutamatergic excitation within the early and ventral visual cortex during viewing of binocular disparity-containing RDS patterns. We show that switching the disparity correlation from correlated to anticorrelated, which removed the visual percept of depth, changed the neurochemistry in these areas: anticorrelated disparity decreased GABA+ and increased Glx in LO, resulting in an increased excitatory drive to anticorrelated disparity. By comparison, in the EVC, anticorrelated disparity decreased Glx. Ultimately, these results suggest that excitation/inhibition balance contributes to solving the stereo correspondence problem along the visual pathway.

### Increases in Glx to correlated disparity in early visual cortex

The primary visual cortex has been the intense focus of research on binocular correspondence. V1 neurons are strongly selective to binocular disparity and remain excited by anticorrelation (Cumming and Parker, 1997). However, the anticorrelated response in V1 is reduced and inverted, pointing toward an early (partial) suppression of false matches. Computational models propose that inhibition at this level suppresses anticorrelation (Read et al., 2002), a process that would reduce feedforward response to false matches at the earliest stage. While we found no change in GABA+, we did find a selective decrease in Glx to anticorrelated compared with correlated stimuli.

Anticorrelated stimuli differ from correlated in several ways: firstly, the low-level matching is different, reflected in V1 neuronal responses; secondly, the correlated stimulus leads to a compelling percept of depth which may have top-down effects on responses; thirdly, anticorrelated stimuli can look like there are more dots, as not all are successfully matched by the visual system (unpublished observation). An increase in Glx during correlated compared with anticorrelated disparity is consistent with a greater neuronal response to correlated disparity (Cumming and Parker, 1997; Janssen et al., 2003). Stereoscopic depth can also engage attentional modulation (Rose et al., 2003; Zou et al., 2022), potentially adding to the net excitation. In summary, the increase in excitation to disparity correlation compared with anticorrelation is consistent with a greater neuronal response to correlated disparity compared with the anticorrelated counterpart that lacks a coherent perceptual solution. The increase in excitation to correlation compared with rest is consistent with findings that show an increase in glutamate with visual stimulation (Mangia et al., 2006; Bednarik et al., 2015; Ip et al., 2017; Kurcyus et al., 2018).

The absence of any modulation during visual stimulation with RDS in EVC GABA+ is consistent with a previous study investigating the metabolic responses to RDS with mixed or single polarity. Rideaux, Goncalves and Welchman (Rideaux et al., 2019) reported no difference in EVC GABA+ between mixed polarity RDS and a baseline condition where subjects had their eyes shut. They also found higher Glx in mixed compared with single polarity RDS, and no difference between mixed polarity and rest. In contrast to their results, we found higher Glx in our equivalent of the mixed condition, the correlated RDS, relative to rest. This difference may be due to the differential use of a rest condition. In their case, participants had their eyes closed, whereas ours fixated on a mid-gray screen. Having eyes open or closed can differentially impact on functional measures (Costumero et al., 2020). The absence of visual stimulation due to having the eyes closed can also increase excitability in the visual cortex (Min et al., 2023), possibly reducing the difference in Glx between baseline and visual stimulation. Overall, these results provide evidence that glutamatergic rather than GABAergic mechanisms may play a role in filtering false matches in the early visual cortex.

### Increases in Glx and decrease in GABA+ to anticorrelation ventral visual cortex

If binocular correspondence in natural vision is already complete before signals arrive in LO, then at the level of LO, signals from false matches would be absent in correlated RDS patterns. Alternatively, if GABAergic inhibition helped filter out false matches in LO, then we might expect to see an increase in GABA+, consistent with the idea of local suppression. Both outcomes would be consistent with strongly suppressed neuronal firing to anticorrelated disparity. Surprisingly, our results showed neither. We found a decrease in inhibition, together with an increase in excitation relative to correlated and rest conditions. This unexpected pattern might suggest that overall, anticorrelation broadly excited neurons in LO. Prior studies have found that anticorrelated stimuli caused less informative (Preston et al., 2008) and decreased hemodynamic response in LO compared with correlated stimuli (Bridge and Parker, 2007; Alvarez et al., 2021; Ip et al., 2022). These population-level measurements contrast with neurophysiological findings that show a complete cessation of response in single neurons (Janssen et al., 2003).

One possibility is that adaptation to anticorrelation over the presentation time of the stimulus caused the balance to shift toward excitation. In support, a prior study has shown an increase in the EEG measure of excitability during presentation of anticorrelated and not correlated disparity (Rideaux et al., 2020). Excitation during the rebound phase of adaptation could thus signal a primary suppressive response to binocular anticorrelation, consistent with the idea that GABAergic inhibition suppresses false matches in the ventral visual cortex. Another possibility is that the increased excitation during anticorrelated disparity presentation relates to perception of number of dots. Indeed, anticorrelated stimuli can sometimes appear like they contain more dots, although the number of dots is constant between correlated and anticorrelated stimuli. This can be explained by a failure to match left and right eye dots during anticorrelation, which can cause a disordered state compared with the binocularly fused percept during binocular correlation (Julesz and Tyler, 1976). In anticorrelated RDS, no coherent object is perceived and therefore LO responds to the individual dots, possibly causing greater excitation. The ventral visual cortex is highly sensitive to numerosity (Paul et al., 2022; Cai et al., 2023), and an increase in cortical excitability could be due to the greater number of dots processed in LO. Future studies can apply multimodal MRI to further establish the mechanism behind disparity processing, for example, by collecting fMRI and MRS simultaneously during binocular disparity processing. In combination, these measures can probe differences in hemodynamics that could relate to changes in neurochemistry. Indeed, simultaneous multi-unit recordings from V1 and V4 in nonhuman primates point toward behaviorally relevant differences in firing pattern between the two areas during binocular depth discrimination (Smith and Parker, 2021). While such studies are rare, they tentatively suggest that fine-grained differences in neuronal computations may underlie the global differences in excitation and inhibition observed in the current study.

### Evidence for robust E/I balance in ventral visual cortex

The balance between excitation and inhibition in the early visual cortex regulates neural processing and plasticity (Hensch, 2005). We assessed the balance between Glx and GABA+ in the early and ventral visual cortex by correlating their concentrations across participants. Initially, Glx and GABA+ correlated in both the early visual cortex and ventral visual cortex. However, controlling for confounding factors, including spectral quality, age, sex, and model fit error demonstrated that the correlation was robust in the ventral but not early visual cortex. Prior studies have shown region specificity of E/I balance, some reporting a balance in the medial parietal cortex (Steel et al., 2020), and others reporting no balance in the visual and motor cortex(Rideaux, 2021). However, a subsequent study by Rideaux et al. (2022) demonstrated E/I balance in the early visual cortex and the discrepancy was suggested to originate from the proxy of excitation used. While using glutamate + glutamine at 3 T may be less reliable than glutamate at 7 T (Rideaux et al., 2022), our results using the 3 T support the existence of E/I balance in the ventral visual cortex of the human brain. This fine balance between excitation and inhibition may increase sensitivity to objects in a manner that is robust to noise (Rubin et al., 2017). This selectivity to complex objects, like faces, may emerge gradually over the course of development (Grill-Spector et al., 2008).

### Object-selective response correlates with Glx during anticorrelated disparity in LO

We found a voxel- and condition-specific correlation between object-selective response and Glx in LO. Our results suggest that individuals who have a higher BOLD response to objects also have more Glx in area LO, specifically during viewing of anticorrelated binocular disparity (Fig. 7*c*). The correlation was specific to LO, robust to controlling for confounding factors including spectral quality, general excitability, and percentage voxel overlap. It is possible that increased Glx during anticorrelation reflects adaptation to the stimulus that would have caused an initial inhibitory response. This hypothesis remains untested in our study but would be consistent with a prior EEG study (Rideaux et al., 2020). Although the stimuli were different in appearance and dimension, greater sensitivity in LO to objects, in general, could explain higher Glx to suppress irrelevant disparity cues, i.e., anticorrelated stimuli, and a stronger response objects. Regressing out EVC anticorrelated Glx concentration values did not weaken the correlation, suggesting that it was not driven by general excitability. This result further points toward excitation in LO as a valuable way to investigate disparity processing.

## Limitations

Our study recruited 18 participants, yet some conditions could not be collected from all participants. In addition, quality control reduced the sample size further, with a minimum of 15 participants per condition. Our test–retest reliability analysis showed differences in correlation coefficients between GABA+ and Glx within voxel locations. We cannot rule out that differences in reliability due to smaller concentrations of GABA+ relative to Glx contributed to the observed pattern. We did not assess depth perception using a behavioral task. This means that the relevance of the Glx and GABA+ changes cannot be directly related to depth perception.

## Conclusion

We measured the concentration of GABA+ and Glx in the early and ventral visual cortex and demonstrated changes in neurochemistry with correlated and anticorrelated binocular disparity. Our results are consistent with previous work suggesting that cortical neurotransmission is involved in suppressing anticorrelated disparity. However, the direction and type of metabolite were not consistent with prior literature. Unlike previously suggested, we found no evidence that GABA inhibited anticorrelated disparity. Instead, we found a decrease in excitation in EVC, suggesting that glutamatergic neurotransmission and metabolism may be involved. In LO, we found an increase in excitation with anticorrelation, possibly reflecting adaptation to prolonged inhibition or more perceived dots in the absence of a coherent percept.

## Synthesis

Reviewing Editor: Niko Busch, Westfalische Wilhelms-Universitat Munster Decisions are customarily a result of the Reviewing Editor and the peer reviewers coming together and discussing their recommendations until a consensus is reached. When revisions are invited, a fact-based synthesis statement explaining their decision and outlining what is needed to prepare a revision will be listed below. The following reviewer(s) agreed to reveal their identity: Jeyoung Jung, Reuben Rideaux.

# Synthesis

This manuscript has been reviewed by two experts in binocular vision and magnetic resonance spectroscopy (MRS). Their comments have been included at the bottom of this letter. Both reviews are positive, highlighting the merits of this study. However, both reviewers are asking for additional information and a few control analyses, and I encourage you to carefully address each of these points. I also support reviewer 2's request to make the data and code publicly available and provide a link to the repository under "Data and Code Accessibility." See for example this paper (under CODE ACCESSIBILITY):

Microsaccades Track Location-Based Object Rehearsal in Visual Working Memory

Eelke de Vries, Freek van Ede. eNeuro 4 January 2024, 11 (1) ENEURO.0276-23.2023; DOI: 10.1523/ENEURO.0276-23.2023

# Reviewer 1

This study investigates the role of GABAergic inhibition and glutamatergic excitation in processing binocular disparity within the human visual cortex, specifically focusing on the distinction between correct and false depth cues. Researchers employed single voxel proton magnetic-resonance spectroscopy (MRS) to measure GABA+ and Glx levels in the early visual cortex (EVC) and lateral occipital cortex (LO) under three conditions: correlated (correct) disparity, anticorrelated (false) disparity, and a control condition.

The findings revealed that in the EVC, correlated disparity led to an increase in Glx, indicating heightened excitatory activity, with a Glx/GABA+ ratio favoring excitation. Conversely, in the LO, anticorrelated disparity resulted in decreased GABA+ and increased Glx, suggesting that false depth cues shift activity toward excitation in the ventral visual stream, which may help suppress these cues.

Overall, this study provides valuable insights into the neurochemical mechanisms underlying depth perception, highlighting the crucial interplay of excitation and inhibition in the visual cortex for accurate stereoscopic vision.

However, I have a few concerns:

• Were the stimuli presented continuously during each MRS condition, without any rest?

• Each condition involved 8 minutes of MRS acquisition. I'm curious whether there were any time-dependent changes in GABA+ and Glx levels. I suggest conducting a sliding-window analysis to explore potential fluctuations in these neurochemicals over time, which could reveal early and transient changes corresponding to the correlated and anticorrelated conditions.

• Was the BOLD activity derived from the object-rest contrast? If so, it might be more informative to correlate BOLD activity with neurochemical levels in each condition relative to rest.

• The authors effectively explained the interaction between the EVC and LO in binocular processing. It might be worthwhile to consider whether functional connectivity between the EVC and LO, as assessed by fMRI, could be linked to neurochemical changes observed in each condition.

# Reviewer 2

Previous computational modelling work suggests that stereopsis may be solved by suppressive mechanisms that inhibit false binocular matches. This study sought to test this hypothesis empirically, by measuring in vivo GABA+ and Glx concentration in the early visual and lateral occipital cortices of human observers while they viewed either correlated or anticorrelated RDS (an additional baseline fixation condition was also include). For EVC, the results showed no differences in GABA between conditions, and increased Glx in the anticorrelated condition, compared to baseline. For LOC, GABA+ was significantly lower in the anticorrelation condition compared to rest, and the ratio of Glx:GABA+ was larger in the anticorrelation condition compared to both correlated and baseline conditions. The researchers additionally found that i) GABA+ and Glx were correlated in LOC, but not EVC, and ii) LOC Glx in the anticorrelated condition was correlated with object-related BOLD responses. These findings are inconsistent with a priori hypotheses and the researchers discuss alternative explanations for the results.

The study clearly structured, meticulously described, and appears to have been conducted rigorously. Although the results are difficult to interpret and somewhat unexpected, they seem robust and will be of particular interest to researchers studying binocular vision, and also visual processing and fMRS more broadly. Hopefully future work can resolve this misalignment between theoretical modelling and empirical measurement. The authors have been quite comprehensive, so I only have a couple of minor requests.

I expect there are differences in the reliability of GABA+ and Glx measurements. That is, the GABA+ signal is probably less reliable than Glx because of its relatively lower concentration. Could the authors compare the correlation between GABA+ and Glx across conditions and within each voxel location (i.e., test-retest reliability)? If GABA+ is less reliable than Glx, this may have obscured differences between conditions (at least in the EVC where none were detected), which is worth acknowledging.

Were there differences in task performance between the conditions? This might speak to differential engagement of attention.

I appreciate the inclusion of Figures displaying the average spectra (which look very good) and voxel overlap, but could these be replotted using only the data that was included in the analyses? That is, remove or separately plot the data that was omitted during outlier rejection.

A previous study found increased GABA+ and decreased Glx in response to single- vs mixed-polarity RDS, but no differences between these conditions and a baseline measurement (Rideaux, Goncalves &Welchman, 2019). It may be appropriate to include a brief discussion of the correspondence between these previous findings and the current results.

I think that it is important that the raw data be made available publicly available on an open database (e.g., osf).

## Author Response

We would like to thank the editor and the two anonymous reviewers for their valuable time and attention. The feedback they have provided has helped us improve our manuscript. Our point-by-point reply is below, including excerpts of the main manuscript that have been revised.

Reviewer 1 1. Were the stimuli presented continuously during each MRS condition, without any rest? Correct, stimuli were presented continuously without any rest. To make this more explicit, we have added the following description to the methods. p. 4 Stimuli were presented continuously during each MRS condition without any rest.

2. Each condition involved 8 minutes of MRS acquisition. I'm curious whether there were any time-dependent changes in GABA+ and Glx levels. I suggest conducting a sliding-window analysis to explore potential fluctuations in these neurochemicals over time, which could reveal early and transient changes corresponding to the correlated and anticorrelated conditions.

We thank the reviewer for the interesting comment and wholeheartedly agree that investigating the time course during continuous presentation is of great value. We quantified dynamic changes in MRS data over time using FSL-MRS and dynamic fitting as it provides a model-driven approach to changes. The same data used in the main analysis also contributed to this control analysis, with the same exclusions applied. The aim was to identify adaptation effects by fitting linear and quadratic models to single-spectrum resolution time courses. Separate analyses were performed for LO and EVC, by condition and metabolite. Results showed no significant model fits for early visual cortex GABA+ or Glx. We also found no significant results for lateral occipital cortex GABA+ or Glx in the correlated condition, apart from a marginally significant result (p=0.05, 5.27% of normalised baseline concentration) for a linear model fit in Glx in the anticorrelated condition. Hence our analysis revealed no consistent evidence for transient fluctuations of neurochemicals over time.

3. Was the BOLD activity derived from the object-rest contrast? If so, it might be more informative to correlate BOLD activity with neurochemical levels in each condition relative to rest.

Thank you for this suggestion. While our BOLD activity to objects was calculated relative to a scrambled object baseline, no fixation only baseline was collected for the BOLD-activity. Also, no comparable baseline was available for the MRS conditions. As clarified in response to the first question, our visual stimuli were presented continuously without rest. We have added the description of our stimulus contrasts to the manuscript, hopefully clarifying what conditions were correlated to each other. p. 11 Using data from the LO voxel, we correlated the 2D object-selective BOLD-responses (intact > scrambled objects) with neurochemistry during continuous viewing of 3D stimuli (Fig. 1c) Therefore, we are not able to equalise the contrast between BOLD and MRS analyses. However, to have any sort of contrast/control in the MRS, we could subtract metabolite concentrations during rest condition from correlated and anticorrelated. Please note that the correlation would still not be matched one-to-one as we would correlate BOLD (objects > scrambled, acquired within one run) with MRS (correlated or anticorrelated > rest, acquired over two independent runs). For this purpose, the Rest condition was subtracted from each stimulation condition in LO for Glx and GABA+, yielding four new variables (LO Glx Corr>Rest, LO Glx Anti>Rest, LO GABA+ Corr > Rest, LO GABA+ Anti> Rest). The difference in metabolite concentration between Stim>Rest was then correlated with the object-selective BOLD-response. No strong evidence was found when stimulus conditions relative to rest were correlated with BOLD-activity (BF10 = 0.41 and below, Figure 1 in this rebuttal letter).

Figure 1 Correlations between object-selective BOLD responses (defined as an intact > scrambled objects contrast) in the Lateral Occipital (LO) cortex and metabolite concentrations in correlated (Corr) and anticorrelated (Anti) conditions relative to the rest condition acquired in an independent run.

4. The authors effectively explained the interaction between the EVC and LO in binocular processing. It might be worthwhile to consider whether functional connectivity between the EVC and LO, as assessed by fMRI, could be linked to neurochemical changes observed in each condition.

We did not collect fMRI measures during anticorrelated and correlated stimulus presentation that could have been used to address this interesting suggestion. There is great value to having complementary measures, such as functional connectivity using fMRI, and we have therefore added this point to our discussion. p. 14 Future studies can apply multimodal MRI to further establish the mechanism behind disparity processing, for example by collecting fMRI and MRS simultaneously during binocular disparity processing. In combination, these measures can probe differences in haemodynamics that could relate to changes in neurochemistry. Indeed, simultaneous multi-unit recordings from V1 and V4 in non-human primates point towards behaviourally relevant differences in firing pattern between the two areas during binocular depth discrimination (Smith and Parker 2021). While such studies are rare, they tentatively suggest that fine grained differences in neuronal computations may underlie the global differences in excitation and inhibition observed in the current study.

Reviewer 2 1. I expect there are differences in the reliability of GABA+ and Glx measurements. That is, the GABA+ signal is probably less reliable than Glx because of its relatively lower concentration. Could the authors compare the correlation between GABA+ and Glx across conditions and within each voxel location (i.e., test-retest reliability)? If GABA+ is less reliable than Glx, this may have obscured differences between conditions (at least in the EVC where none were detected), which is worth acknowledging.

We compared the correlation between GABA+ and Glx across conditions and within each voxel location for test-retest reliability. The same data used in the main analysis also contributed to this control analysis, with the same exclusions applied. We split each run into the first half and second half, consisting of 80 difference spectra each. These were preprocessed and fit separately. Metabolite concentrations were then averaged across correlated, anticorrelated and rest conditions. The Pearson's correlation coefficient between the first and second half by metabolite (GABA+, Glx) and by the voxel location (EVC, LO) was calculated as a measure of reliability. Finally, a two-tailed Fisher r-to-z transform test was used to evaluate the significance of the difference between correlation coefficients across conditions and within each voxel location. There was a significant difference in correlation coefficients between GABA+ and Glx for the LO voxel (p = 0.001), and a marginally significant difference for the EVC voxel (p = 0.03). As the reviewer pointed out, this is consistent with the lower concentration of GABA+ relative to Glx. Importantly, there was no difference between voxels for either GABA+ (p = 0.6) or Glx (p = 0.6). Overall, correlation coefficients were very high, at r = 0.80 or above for GABA+ and above r = 0.97 or above for Glx (see Table 1 below).

Table 1. Pearson's correlation coefficients between the first and second half of the scan for each metabolite and voxel location, averaged across conditions.

Hence, we found no evidence that GABA+ or Glx differed in test-retest reliability between voxels. Test-retest reliability is unlikely to have obscured effects in the EVC.

2. Were there differences in task performance between the conditions? This might speak to differential engagement of attention.

Unfortunately, behavioural data were not collected during the task due to a Matlab software error. In an almost identical fixation task performed during presentation of correlated and anticorrelated disparity (xxxxxx et al., 2024, in review), we found no differences in percentage correct between the two conditions (correlated: 69 {plus minus} 9 %, anticorrelated: 75 {plus minus} 6 % correct, two-tailed paired samples T-test, p = 0.5). This suggests that the disparity content did not differentially engage attention. The following clarification has been added to our methods. p. 5 A similar task was used in xxxxxx et al., (unpublished), where there was no difference in performance between correlated and anticorrelated stimuli. In the current study we did not record the button presses.

3. I appreciate the inclusion of Figures displaying the average spectra (which look very good) and voxel overlap, but could these be replotted using only the data that was included in the analyses? That is, remove or separately plot the data that was omitted during outlier rejection.

Thank you for your positive feedback on the average spectra. To make the average spectra more representative, we have replotted the figures, excluding the datapoints flagged in the outlier rejection procedure for GABA+. d) Group MRS spectra for each condition and voxel, including only data that was used for the main GABA+ analysis. The black line represents the mean across participants, the grey area shows {plus minus}1 standard deviation across participants and the red line shows the model fit.

We have also added the methods for constructing the average spectra to our methods. p. 6 Group spectra were constructed using fsl_mrs_summarise. Only data used for GABA+ analysis, after outlier rejection, were included in group spectra (Fig. 2d).

The voxel overlap data is already representative, as all the participants contributed to one of the conditions in the EVC or LO analyses and has not been changed.

4. A previous study found increased GABA+ and decreased Glx in response to single- vs mixed-polarity RDS, but no differences between these conditions and a baseline measurement (Rideaux, Goncalves &Welchman, 2019). It may be appropriate to include a brief discussion of the correspondence between these previous findings and the current results.

We are aware of this relevant paper by Rideaux, Goncalves and Welchman, 2019, which investigated the metabolic activity underlying the mixed-polarity advantage. The main difference between our studies is that all our stimuli were of 'mixed polarity', containing equal numbers of black and white dots. The comparable conditions across our studies are hence the 'correlated' and 'anticorrelated' vs 'rest' conditions, leaving out their main comparison of interest of single polarity. Because of this discrepancy we can only discuss the aspects that we have in common. p. 13 The absence of any modulation during visual stimulation with RDS in EVC GABA+ is consistent with a previous study investigating the metabolic responses to RDS with mixed or single polarity. Rideaux, Goncalves and Welchman (Rideaux et al., 2019) reported no difference in EVC GABA+ between mixed polarity RDS and a baseline condition where subjects had their eyes shut. They also found higher Glx in mixed compared to single polarity RDS, and no difference between mixed polarity and rest. In contrast to their results, we found higher Glx in our equivalent of the mixed condition, the correlated RDS, relative to rest. This difference may be due to the differential use of a rest condition. In their case, participants had their eyes closed, whereas ours fixated on a mid-grey screen. Having eyes open or closed can differentially impact on functional measures (Costumero et al., 2020). The absence of visual stimulation due to having the eyes closed can also increase excitability in the visual cortex (Min et al., 2023), possibly reducing the difference in Glx between baseline and during visual stimulation.

5. I think that it is important that the raw data be made available publicly available on an open database (e.g., osf).

We agree with the reviewer that public availability of the data is important. The full raw dataset, including anatomical images, MRS data and BOLD-fMRI data has been uploaded and will be made public upon acceptance of the manuscript using the URL in the manuscript. The exact address has been removed to maintain the anonymity of the authors. p. 5 The raw MRI data can be accessed at https://zenodo.org/records/xxxxx
